# Land use/land cover dynamics driven changes in woody species diversity and ecosystem services value in tropical rainforest frontier: A 20-year history

**DOI:** 10.1016/j.heliyon.2023.e13711

**Published:** 2023-02-14

**Authors:** Yericho Berhanu, Gemedo Dalle, Dejene W. Sintayehu, Girma Kelboro, Abebe Nigussie

**Affiliations:** a Africa Center of Excellence for Climate Smart Agriculture and Biodiversity Conservation, Haramaya University, Ethiopia; b Department of Natural Resource Management, College of Agriculture and Natural Resources, Bonga University, Ethiopia; c Center for Environmental Science, Addis Ababa University, Addis Ababa, Ethiopia; d College of Agriculture and Environmental Sciences, Haramaya University, Ethiopia; e Center for Development Research (ZEF), University of Bonn, 53113, Bonn, Germany; f Department of Natural Resource Management, College of Agriculture and Veterinary Medicine, Jimma University, Ethiopia

**Keywords:** Biodiversity, Conservation, Deforestation, Landscapes

## Abstract

Land use/land cover (LULC) change is a prominent problem in tropical forests. However, the fundamental question of how much woody species diversity was lost and ecosystem services value (ESV) changed in response to LULC conversion has rarely been studied. Therefore, the objective of this study was to assess the impact of LULC change on woody species diversity and ecosystem service value in the last two decades in the tropical rainforest frontier taking the case of Sheka Forest Biosphere Reserve (SFBR), Southwest Ethiopia. Supervised image classification with a maximum likelihood approach was employed and 90 quadrants were laid for the woody species inventory. Diversity indices and descriptive statistics were computed and the non-parametric test (Kruskal-Wallis) was used to test the effect of LULC change on woody species diversity. The benefit transfer method was used to estimate the monetary value of ecosystem services adopting coefficients from empirical studies. The woody species richness, diversity, and evenness varied (X^2^ = 71.887, p < 0.05) across LULC types. The highest diversity was observed in the forest followed by cropland, coffee plantation, homegarden, and tea plantation. The estimated total ecosystem service value (ESV) was reduced by 21.56% from 309.11 million US$ in 1999 to 242.47 million US$ in 2020. Transition to mono-crop like tea plantations to maximize income not only altered native woody species but also induced the expansion of exotic species and reduced ESV, indicating a detrimental impact of LULC change on ecosystem integrity and stability in the future. Although LULC conversion destruct woody species diversity, cropland, coffee plantation and the homegardens were the refuge for some endemic and conservation priority species. Further, addressing contemporary challenges of LULC conversion through introducing mechanisms such as payment for ecosystem services that increase the economic and livelihood benefits of natural forests to local communities is important. Effective conservation and sustainable use approaches in which such species are systematically integrated into land use practices have to be planned and implemented. This could contribute to strengthening the conservation effectiveness of the SFBR of UNESCO and serve as a showcase for such conservation areas around the globe. The LULC challenges, particularly those emanating from local livelihood needs, could impede our efforts to conserve biodiversity, jeopardize the reliability of future projections, and have an impact on the conservation of threatened ecosystems, if it is not adequately addressed in time.

## 1 Introduction

Land use/land cover (LULC) change is a critical challenge in tropical forests due to its detrimental effect on the environmental quality and the availability of resources [1]. Accelerated LULC change caused by rapid population growth, agricultural land, and settlement expansions [[2], [3], [4], [5], [6]] imposed environmental degradation, biodiversity loss, and changes in ecosystem service value [7,8]. The expansion of agricultural land at the expense of natural forest is the typical phenomenon of LULC change in the tropical forests of Ethiopia [9]. Forestland has been leased to agricultural investment and the demand to change the remaining natural forest to agricultural land is prominent in the tropical rainforest frontier in Ethiopia [10]. Forestland conversion to other land use types could have a detrimental impact on biodiversity and associated ecosystem service values [11]. Although LULC change is ubiquitous, the impacts of LULC change on the diversity of woody species and ecosystem services in tropical forest frontiers were not sufficiently studied.

LULC change analysis was used as a technique to comprehend the relationship between humans and nature [12]. In this regard, numerous studies detected LULC change and identified trends, patterns, and consequences of deforestation in general [4,[13], [14], [15], [16], [17], [18], [19], [20]] and tropical rainforest of global biosphere reserves of Ethiopia in particular [21]. However, the previous studies did not show LULC conversion-driven changes in woody species diversity and associated ecosystem services value change in Forest Biosphere Reserve in Ethiopia. As the result, the basic question of how much woody species diversity has been lost and ecosystem service (ES) value changed in response to LULC conversion remains largely unaddressed in the global biosphere reserves of Ethiopia, and most land use management decisions have failed to take this into account in the area.

The Sheka Forest Biosphere Reserve (SFBR) is one of five biosphere reserves in Ethiopia [22]. It is part of Eastern Afromontane biodiversity hotspots and is distinctly characterized by encompassing a few remnant tropical rainforests [23]. SFBR is among the top national forest priority areas in Ethiopia. Previously, the landscape of SFBR was managed and conserved by the community with their indigenous knowledge, beliefs, and culture through generations [24]. The indigenous knowledge of species and forest management practices of the local community contributed to the existence of a few remnant Afromontane forests [25]. Since, 2012 Sheka forest has been designated as a global biosphere reserve with aim of reversing deforestation and degradation to enhance biodiversity conservation and associated ecosystem service value [26]. However, persistent LULC change trajectories [10] might lead to the loss of woody species diversity and the degradation of ecosystem services. The extent to which designating the forest as a biosphere reserve, the program under implementation since 2012, prevents forestland conversion to other uses and saves biodiversity and ecosystem services are questionable.

Previously, numerous studies were conducted in moist Afromontane forests of Southwest Ethiopia in general [[27], [28], [29], [30]] and SFBR in particular [31]. Most of these studies quantified the status of wood plant species diversity in natural forests. Some studies conducted comparative analyses on the variation of woody species between managed and disturbed forests including coffee agroforestry systems [28,32]. The previous studies pinpoint the urgency of biodiversity conservation through natural forest preservation [33,34]. Although a few studies attempted land-use legacy on woody vegetation in Southwest Ethiopia [35], most of the previous studies missed to explore the impacts of LULC changes on woody species diversity and ecosystem service value.

The absence of scientific accounts to show the trade-off between LULC conversions and biodiversity loss and ecosystem services value changes also contributed to the frail decision to convert land uses in the area. On the other hand, evaluation of the trade-offs and synergies between LULC change and biodiversity conservation as well as associated ecosystem services value change is of paramount importance and serves as good evidence for setting appropriate conservation and management strategies [36]. Therefore, the specific objectives of this study were (1) to assess the impact of LULC change on woody species diversity in comparison with adjacent natural forests, and (2) to estimate LULC dynamics-driven changes in ecosystem service value for the last two decades (1999–2020) in tropical rainforest frontier taking the case of Sheka Forest Biosphere Reserve, Southwest Ethiopia. The change in ecosystem service value due to LULC change from 1999 to 2020 was estimated using the modified ecosystem service value coefficients and the benefits transfer valuation method. Therefore, we hypothesize that the LULC change has caused a loss in woody species diversity and a reduction in the value of ecosystem services in SFBR for the last two decades.

## 2 Materials and methods

### 2.1 Description of the study area

**Fig. 1:**
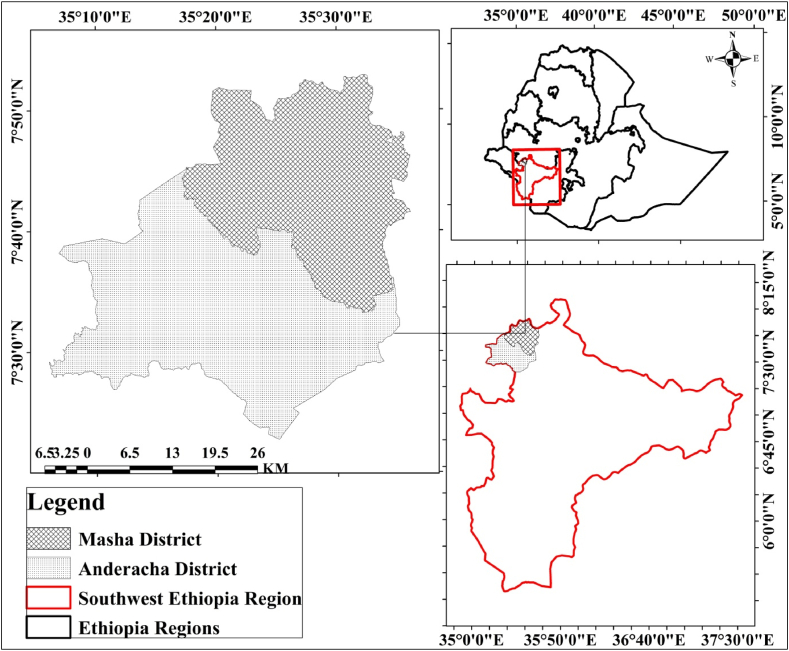
Study area map.

This study was conducted on SFBR in the Masha and Anderacha districts of Southwest Regional State, Ethiopia. Since 2012, SFBR has been designated and included in the global biosphere reserves network by the MAB program of UNESCO [26]. The SFBR is located between 07° 10′ to 7° 55′ N latitude and 35° 10 to 35° 50′ E longitude with a total landmass of 2382.61 km^2^ (Fig. 1).

The SFBR encompasses forests, bamboo thickets, wetlands, agricultural land, rural settlements, and small towns. The forest of SFBR is classified as a moist evergreen montane forest with a floristic composition of Transitional Rainforest, Broad-leaved Afromontane Rainforest, and Riverine Forest [23]. Farming, beekeeping, and livestock rearing are the mainstay of the community’s livelihood. Following the expansion of tea and coffee plantations and urbanization, the selling of forest products like timber, lumber, lianas, firewood, and charcoal has become the source of income in the area.

### 2.2 Data sources

**Fig. 2:**
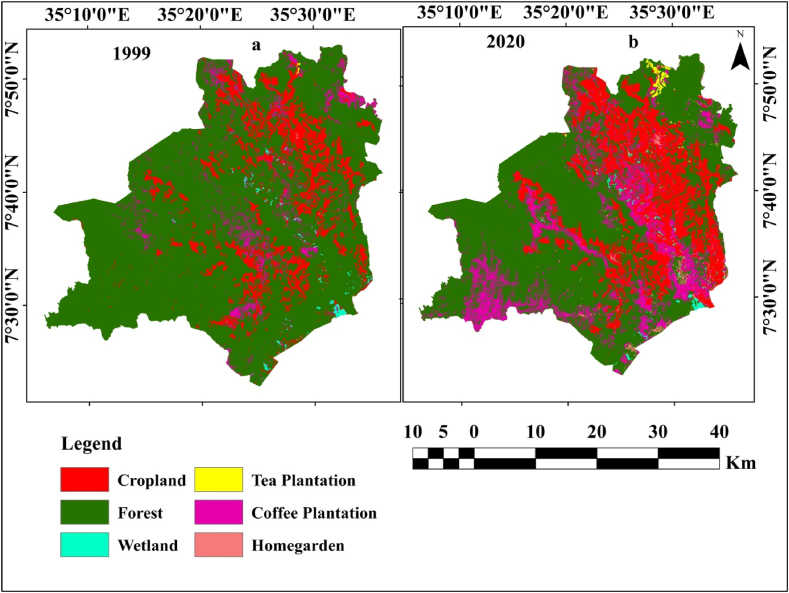
Spatial distribution map of each LULC type for 1999 (a) and 2020 (b).

The LULC data for the years 1999 and 2020 was obtained from Ref. [10] and the spatial distribution map of each LULC type is presented in Fig. 2.

The study identified six LULC types (cropland, forest, coffee plantation, tea plantation, wetland, and homegarden) using a supervised classification algorithm following the maximum likelihood classification approach (Table 1). The ecosystem service value (ESV) coefficients for targeted LULC types were obtained from empirical studies [11,36,37] and presented in (Table 1).

**Table 1:** Spatial coverage of LULC types in 1999 and 2020 with corresponding equivalent biomes and ESV coefficient.

LULC	Spatial coverage (ha)		Change (%)	Equivalent biome	ESV coefficient (US $ha^−1^ yr^−1^)
	1999	2020	1999 to 2020		
FL	145,264	100,121	−31.1	Agriculture	92
CL	23,250	41,968	80.5	Tropical forest	2007
CP	8460.1	32,377	282.7	Shrub land	986.69
TP	43.7	1127.5	2478	Agriculture	92
Hg	91.4	1695.8	1755	Urban	0
WL	872.6	692.7	−20.6	Rivers/Lakes	8103.5

FL stands for forest land, CL for cropland, CP for coffee plantation, TP for tea plantation, Hg for settlement (Home garden), and WL for the wetland.

### 2.3 Sampling design

Out of six LULC types identified in the study area; cropland, forest, coffee plantation, tea plantation, and home garden were stratified for woody species inventory. Wetland was not considered for woody species diversity assessment since it was rarely covered by woody species [38,39]. After stratification, sample land uses were selected randomly from all LULC types. The adjacent natural forests that have remained as forests for the last two decades were used as control. Then, three transect lines that possess 18 quadrants were laid for each LULC type. This resulted in a total of 90 quadrants for the five LULC types. The distance between transect lines was 300 m and the interval between sample plots along the transect line was 300 m as used by Ref. [40]. The vegetation inventory was employed in the quadrants with the dimension of 10 m × 50 m.

### 2.4 Data collection

In each sample plot, all individual trees, shrubs, and lianas with a height above 2 m were counted and recorded. The diameter and height of individual woody species were measured. The diameter was measured at breast height for the tree (DBH) and stump height (DSH) for the shrub with DBH/DSH above 2.5 cm. The caliper and diameter tape were used for diameter measurement. If the tree branched at breast height, the diameter was measured separately for the branches. In cases where tree boles were buttressed, diameter measurements were made just above the buttresses.

The height of every individual woody species having DBH/DSH greater than 2.5 cm was measured by the SUUNTO clinometer. The species identification was done in the field based on vernacular names using supplementary field guidebooks [[41], [42], [43], [44], [45], [46]] and indigenous knowledge of woody species by local people. For the cases in which the identification was difficult in the field, the specimen was collected and taken to Haramaya University Herbarium for identification.

### 2.5 Vegetation composition and structure analysis

The woody species genera and families were thoroughly identified and quantified using descriptive statistics. The vegetation structure was analyzed by computing mean DBH, mean height, density, and basal area. The basal area (Eq. (1)) and density (Eq. (3)) were computed on a hectare basis.

(1) $$Basal\;area=\frac{\pi {DBH}^{2}}{4}$$

The Importance Value Index (IVI) was computed to determine the vegetation structure and the dominant species using Eqs. (2), (3), (4), (5)).

(2) IVI=RD+RF+RDo

(3) $$RD=\left(\frac{Number\;of\;individuals\;of\;species}{Total\;number\;of\;individuals\;of\;all\;species}*100\right)$$

(4) $$RF=\left(\frac{Frequency\;of\;a\;species}{Sum\;of\;frequencies\;of\;all\;species}*100\right)$$

(5) $$RDo=\left(\frac{Basal\;area\;of\;a\;single\;species}{Total\;basal\;area\;of\;all\;species}*100\right)$$

where; RD is relative density, RF is relative frequency, and RDo is Relative dominance.

The Jaccard and Sorensen index (Eq. (6)) was calculated to evaluate the similarity of the woody species composition of different land-use types [47].

(6) $$S=\frac{2A}{2A+B+C}$$

where; S is the similarity index, A is the number of species common in two LULC types, B is the number of species present only in LULC type B, and C is the number of species present only in LULC type C.

### 2.6 Species richness, diversity, and evenness analysis

Species diversity will be measured by using both species richness and evenness indexes. Species richness is the number of species within a biological community and computed using Eqs. (7), (8)). Evenness is the distribution of individuals among species [48] and computed using Eq. (9).

(7) $$pi=\frac{xi}{\sum_{i=1}^{s}xi}$$

where, pi is a proportional abundance of species i, xi is the abundance of species

(8) $$H^{\prime }=-\sum_{i=1}^{s}pi\;\log\;pi$$

*where H*′ *is Shannon-Wiener Index.* The minimum value of *H*′ is zero when only one species is present in the area and the maximum value is log(S) when all species have equal abundances. S is the number of specific types.

(9) $$J=\frac{-\sum_{i=1}^{s}pi\;\log\;pi}{\mathrm{Log}\left(S\right)}$$

*where J is Pielou*’*s index. J* has the maximum value of 1 when species have equal abundances and approaches zero as one species dominates.

### 2.7 Ecosystem service value estimation

The ES values were estimated using the benefits transfer method. This approach is widely used to estimate the ESV in the absence of site-specific valuation information [8,49,50]. In this study, the ES value estimation was based on the LULC of the study area and global ES value from the database, and the total value of ES was computed using Eq. (10).

(10) $$ESV=\sum_{1}^{i}\left(A_{k}*{VC}_{k}\right)$$

where ESV is the total ecosystem service value, A_k_ is an area (ha) and VC_k_ is the coefficient value (US$ ha^−1^ yr^−1^) for LULC category k and i is the number of LULC types. The value of individual ecosystem services was estimated using Eq. (11).

(11) $${ESV}_{f}=\sum \left(A_{k}*{VC}_{fk}\right)$$

where ESV_f_ is the estimated ecosystem service value of function f and VC_fk_ is the coefficient value (US$ ha^−1^ yr^−1^) for function f for LULC category k.

The ESV change over time was computed by using Eq. (12).

(12) $$ESV\;change\left(\%\right)=\frac{\left(ESV\;of\;final\;year-ESV\;intial\;year\right)}{\left(ESV\;intial\;year\right)}*100$$

A sensitivity analysis was carried out to determine the potential change in ESVs for change in the value coefficient [9]. When a variable changes with other changes, it is said to be elastic [11]. The elasticity of the ESV change was therefore used to calculate the % change in ESV in response to changes in LULC in SFBR. The modified ESV coefficients for targeted LULC types were adjusted by 50% and the coefficient of sensitivity was computed using Eq. (13) as used by Ref. [9].

(13) $$CS=\frac{{ESV}_{j}{-ESV}_{i}{/ESV}_{i}}{{VC}_{jk}{VC}_{ik}{/VC}_{ik}}$$

where CS = Coefficient of Sensitivity, ESV_i_ is the initial adjusted total ESV, ESV_j_ is the adjusted total ESV. VC_ik_ and VC_jk_ are the initial and adjusted value of the coefficient (US$ ha^−1^ yr^−1^) for LULC type k, respectively.

In this study, the elasticity of change in ESV was used to evaluate the fairness of valuation in our analysis.

### 2.8 Data analyses

Descriptive statistics were used for the comparison of woody plant diversity among different land-use types. The underlying assumption of the normality distribution was examined by plotting the Q-Q plot and histogram and tested using Bartlett’s test. Yet, the normality assumption of data distribution was not sufficiently maintained in the observed data set. Therefore, the non-parametric test (Kruskal-Wallis) was used to test the effect of LULU change on woody species diversity. Tukey’s HSD test was employed for mean separation. The analysis was performed using R statistical software (version 4.1.0) (R Core Team, 2021).

## 3 Results

### 3.1 Woody species composition

A total of 84 woody species that belong to 73 genera and 39 families were recorded from 90 quadrants in the five land-use types in the area. About 40.5% of woody species were from six families with the share of Rubiaceae (9.52%), Fabaceae (8.33%), Celastraceae (5.95%), Euphorbiaceae (5.95), Malvaceae (5.95%), and Moraceae (4.76). Araliaceae, Oleaceae, Rosaceae, Sapindaceae, and Ulmaceae share 17.86% of the total woody species each of them was represented by three species. Furthermore, having two species each, the Acanthaceae, Asteraceae, Boraginaceae, Dracaenaceae, Myrtaceae, Rhamnaceae, and Rosaceae families accounted for 16.67% of total woody species. With one species each, the other 21 families accounted for 25% of woody species (Table 2).

**Table 2:** The woody families recorded with their number and percentage (share of total) of genera and species in SFBR.

Family	Species		Genus		Family	Species		Genus	
	Number	%	Number	%		Number	%	Number	%
*Rubiaceae*	8	9.52	8	10.96	*Aquifoliaceae*	1	1.19	1	1.37
*Fabaceae*	7	8.33	6	8.22	*Asclepiadaceae*	1	1.19	1	1.37
*Euphorbiaceae*	5	5.95	5	6.85	*Combretaceae*	1	1.19	1	1.37
*Malvaceae*	5	5.95	5	6.85	*Cupressaceae*	1	1.19	1	1.37
*Celastraceae*	5	5.95	3	4.11	*Cyatheaceae*	1	1.19	1	1.37
*Moraceae*	4	4.76	2	2.74	*Icacinaceae*	1	1.19	1	1.37
*Oleaceae*	3	3.57	3	4.11	*Lauraceae*	1	1.19	1	1.37
*Araliaceae*	3	3.57	2	2.74	*Melianthaceae*	1	1.19	1	1.37
*Rosaceae*	3	3.57	2	2.74	*Myrsinaceae*	1	1.19	1	1.37
*Sapindaceae*	3	3.57	2	2.74	*Palmae*	1	1.19	1	1.37
*Ulmaceae*	3	3.57	2	2.74	*Phytolaccaceae*	1	1.19	1	1.37
*Acanthaceae*	2	2.38	2	2.74	*Pittosporaceae*	1	1.19	1	1.37
*Boraginaceae*	2	2.38	2	2.74	Proteaceae	1	1.19	1	1.37
*Myrtaceae*	2	2.38	2	2.74	*Rhizophoraceae*	1	1.19	1	1.37
*Rhamnaceae*	2	2.38	2	2.74	*Sapotaceae*	1	1.19	1	1.37
*Rutaceae*	2	2.38	2	2.74	*Simaroubaceae*	1	1.19	1	1.37
*Asteraceae*	2	2.38	1	1.37	*Sterculiaceae*	1	1.19	1	1.37
*Dracaenaceae*	2	2.38	1	1.37	Theaceae	1	1.19	1	1.37
*Anacardiaceae*	1	1.19	1	1.37	*Vitaceae*	1	1.19	1	1.37
*Apocynaceae*	1	1.19	1	1.37	Total	84	100	73	100

The analysis of the genera showed that Rubiaceae is the most dominant in the area having 8 genera (9.96%) followed by Fabaceae with 6 genera (8.22%). Euphorbiaceae contributed 5 genera (6.84%) and Malvaceae 5 genera (5.5%). The growth habit recorded indicated that 77.5% of woody species belong to the tree, 19% to shrubs, and 3.5% to lianas.

### 3.2 Height, DBH, density, and basal area

**Fig. 3:**
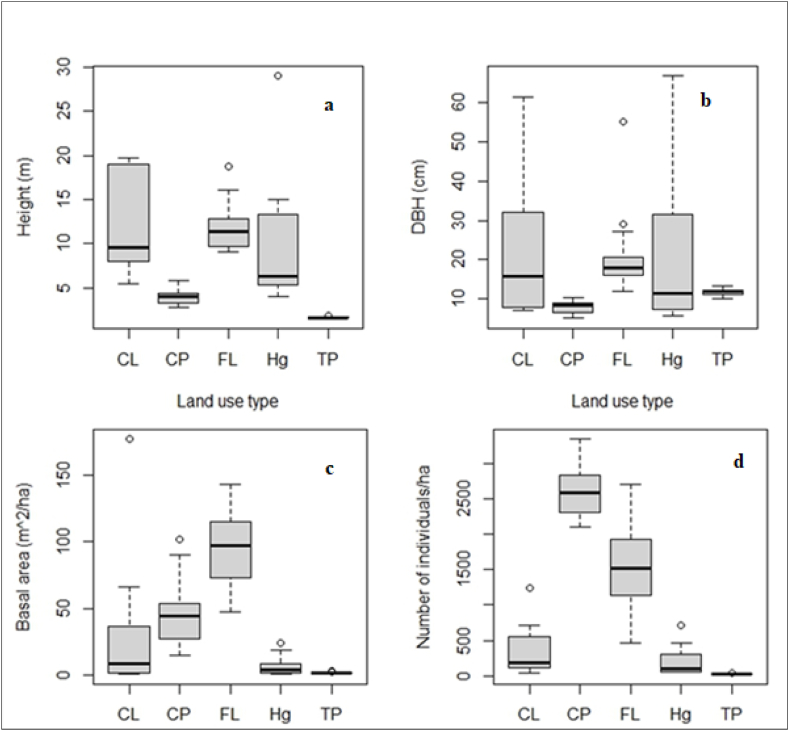
Mean height (>2 m) (a), mean DBH (DBH >2.5 cm) (b), Basal area (c), density (d) by land use type in SFBR. Abbreviation as in Table 1.

The number of woody stems under a given height and DBH class in LULC type was presented in Table 3. The woody species density was highest in coffee plantations (2612 ± 87 individual ha^−1^) and followed by forests (1476 ± 140 individual ha^−1^) (Table 3; Fig. 3). The woody species density of cropland (332 ± 72 individual ha^−1^) was lower than forest, while higher than a home garden (194 ± 44 individual ha^−1^). The lowest woody species density was recorded in tea plantations (23 ± 3 individual ha^−1^). The average woody species density of coffee plantations was significantly higher than forests (p < 0.05). However, the average woody species densities in cropland, home garden, and tea plantations were significantly lower than in forests (p < 0.05).

**Table 3:** Height and DBH class frequency distribution (number of individual ha^−1^) in LULC type in SFBR.

	Class	FL	CP	CL	Hg	TP
Height	2–6 m	862	2364	207	137	0
	6.01–10 m	366	41	47	50	0
	>10 m	248	207	79	8	23
	Total	1476	2612	332	194	23
DBH	2.5–10 cm	672	2333	227	121	0
	10.1–20 cm	488	47	32	61	0
	20.1–30 cm	133	113	21	0	0
	30.1–40 cm	38	48	30	2	23
	40.1–50 cm	44	19	7	0	0
	>50 cm	100	52	16	10	0
	Total	1476	2612	332	194	23

Abbreviation as in Table 1.

The average height, DBH, basal area, and density of woody species (the height above 2 m and BDH above 2.5 cm) were presented in Fig. 3. The average height of woody species was highest in the forest (11.9 ± 0.6 m) and cropland (11.5 ± 1.3 m) followed by the home garden (8.7 ± 1.5 m), and coffee plantation (3.9 ± 0.2 m), while lowest in tea plantation (1.6 ± 0.1 m). The average DBH of woody species was highest in cropland (24.2 ± 4.5 cm) followed by forest (20.62 ± 2.24 cm) and home garden (20.62 ± 4.48 cm), while lowest in coffee (7.81 ± 0.39) and tea plantations (11.73 ± 0.23 cm) (Fig. 3).

The average basal area was 94.79 ± 8.88 m^2^ ha^−1^for forests, 46.4 ± 4.92 m^2^ ha^-1^ for coffee plantations, 22.83 ± 8.461 m^2^ ha^1^ for cropland, 5.86 ± 1.44 m^2^ ha^-1^ for the home garden, and 1.58 ± 0.1 m^2^ ha^−1^for tea plantations (Fig. 3). The statistical test result shows that the basal area of coffee plantations, cropland, home garden, and tea plantations was significantly lower than the basal area of forest (Table 4).

**Table 4:** Woody species density and basal area across land use type.

Variables	LULC type	Estimate	*Chi-square*	P value
Density (individual ha^−1^)	FL	1476 ± 140^a^	79.928	0.000∗
	CP	2612 ± 87^b^		
	CL	332 ± 72^c^		
	Hg	194 ± 44^c^		
	TP	23 ± 2^d^		
Basal area (m^2^ha^−1^)	FL	94.79 ± 8.88^a^	62.119	0.000∗
	CP	46.4 ± 4.92^b^		
	CL	22.83 ± 8.461^c^		
	Hg	5.86 ± 1.44^cd^		
	TP	1.58 ± 0.1^d^		

∗ denote significance at a 95% level of confidence, and different superscript letters denote significant differences among group means. Abbreviation as in Table 1.

### 3.3 Importance Value Index (IVI)

The IVI of woody species was computed and the results were presented (Appendix I). The most dominant woody species in the natural forest were *Schefflera myriantha* (Bak.) Drake, *Syzygium guineense* (Willd.) DC, *Macaranga capensis* (Baill.) Sim, *Croton macrostachyus* Del, and *Ficus vasta* Vahl with IVI values of 28.19, 17.60, 13.11, 12.85, and 12.30 respectively. On the other hand, *Vepris dainellii* (Pich.-Serm.) Kokwaro, *Celtis gomphophylla* Bak, *Phytolacca dodecandra* L’Hérit, *Deinbollia kilimandscharica* Taub, and *Ehretia abyssinica* R. Br. ex Fresen were the rarest woody species with the corresponding IVI of 0.58, 0.76, 0.76, 0.82, and 0.90 respectively.

The IVI result shows that *Coffea arabica (117.11)*, *Millettia ferruginea* (Hochst.) Bak (31.69), *Cordia africana* Lam (27.23), *S. abyssinica* (23.75), and *Albizia gummifera* (Gmel.) C. A. Sm (14.82) was the most dominant woody species in coffee plantations in coffee plantations. *Euphorbia ampliphylla* Pax*, S. guineense, Maytenus gracilipes* (Welw. ex Oliv.) Exell*, Schefflera abyssinica* (Hochst. ex A. Rich.) Harms*,* and *Croton macrostachyus* Del. were dominant woody species in cropland with the corresponding IVI values of 38.27, 38.18, 37.19, 36.31, and 17.21 respectively. Tea plantation is mono-cropping, while *Grevillea robusta* was planted for hedge.

### 3.4 Species similarity

The woody species similarity across LULC types was computed and presented below in Table 5. The result shows that there was a high similarity of woody species between forest and cropland (40%) followed by forest and coffee plantations (35%). Furthermore, the highest (44%) woody species similarity was observed between coffee plantations and home gardens.

**Table 5:** Similarity of woody species across LULC type in SFBR.

LULC types	Similarity index
FL versus CP	0.35
FL versus CL	0.40
FL versus Hg	0.08
FL versus TP	0
CP versus CL	0.41
CP versus Hg	0.44
CP versus TP	0.06
CL versus Hg	0.39
CL versus TP	0
Hg versus TP	0

Abbreviation as in Table 1.

### 3.5 Species richness, diversity, and evenness

**Fig. 4:**
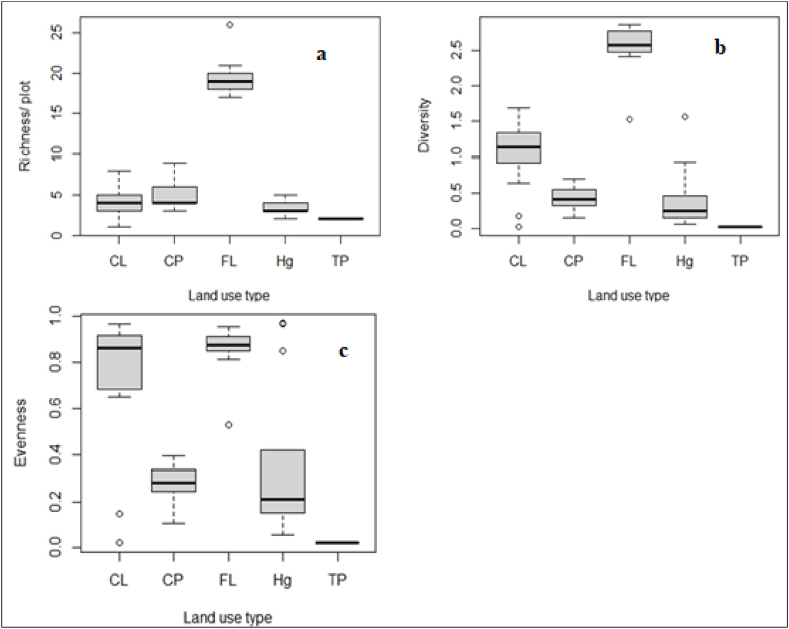
Box plot showing variation in woody species richness (a), diversity (b), and evenness (c) among land-use types. Abbreviation as in Table 1.

The woody species' richness, diversity, and evenness across different land use were presented in Fig. 4, and Table 6. The number of woody species significantly varies by land-use type (Table 6). The result shows that species richness was highest in the forest followed by coffee plantations and cropland. Tea plantations had the lowest species richness.

**Table 6:** Mean of species richness, diversity, and evenness and statistical test results by LULC type.

Variables	LULC type	Estimate	Chi-square	P value
Species richness	FL	62^a^	69.433	0.000∗
	CP	29^b^		
	CL	29^b^		
	Hg	12^c^		
	TP	2^d^		
Species diversity	FL	2.51 ± 0.090^a^	71.887	0.000∗
	CP	0.427 ± 0.034^b^		
	CL	1.029 ± 0.123^c^		
	Hg	0.364 ± 0.088^d^		
	TP	0.014 ± 0.000^e^		
Species evenness	FL	0.845 ± 0.029^a^	59.132	0.000∗
	CP	0.279 ± 0.019^b^		
	CL	0.727 ± 0.075^c^		
	Hg	0.326 ± 0.070^d^		
	TP	0.021 ± 0.000^e^		

∗ denote significance at a 95% level of confidence, and different superscript letters denote significant differences among group means. Abbreviation as in Table 1.

Woody species diversity was highest in the forest followed by cropland and home garden. However, woody species diversity was lowest in tea plantations followed by coffee plantations. The statistical test confirms that the woody species diversity significantly varies across LULC types in SFBR.

### 3.6 Endemic species and conservation priority

A total of three endemic species namely (i) *Erythrina brucei* Schweinf, (ii) *Millettia ferruginea* (Hochst.) Bak, and (iii) *Vepris dainellii* (Pich.-Serm.) Kokwaro were recorded across landscapes in SFBR (Table 7). The distribution of the observed endemic varies across different land-use types. The highest number of endemic species was encountered in forests and croplands, while not observed in tea plantations.

**Table 7:** Distribution of Endemic plant species across different land-use types in SFBR.

Scientific name	LULC type				
	FL	CP	CL	Hg	TP
*Erythrina brucei* Schweinf.	x	–	x	–	–
*Millettia ferruginea* (Hochst.) Bak	–	x	x	x	–
*Vepris dainellii* (Pich.-Serm.) Kokwaro	x	–	–	–	–

Abbreviation as in Table 1, X stands for species occurrence, while – stands for non-occurrence*.*

Furthermore, 22 woody species that were not observed in the forests were encountered in the other land use types (six in coffee plantations, five in cropland, one in the home garden, one in the tea plantation, and nine shared by two or more land-use types).

### 3.7 Changes in ecosystem services value

The total ESV was 309 million US$ in 1999 and 242.47 million US$ in 1999. The total ESV was reduced by 21.56% for the last two decades in SFBR (Table 8). The decline of forests and wetlands resulted in the total ESV reduction in the study area.

**Table 8:** Estimated total ecosystem service value and change from 1999 to 2020.

LULC type	ESV (US$ million)		ESV change (US$ million)	
	1999	2020	1999–2020	%
CL	2.14	3.86	1.72	80.51
FL	291.54	200.94	−90.60	−31.08
CP	8.35	31.95	23.60	282.70
TP	0.00	0.10	0.10	2480.09
WL	7.07	5.61	−1.46	−20.62
Hg	0.00	0.00	0.00	–
Total	309.11	242.47	−66.64	−21.56

Abbreviation as in Table 1.

Of the four main groups of ES category, regulating service had the highest ESV accounting for 151.34 million US$ in 1999 and 119.25 million US$ in 2020, followed by provisioning with ESV of 103.97 million US$ and 80.85 million US$, respectively. The lowest ESV was recorded in supporting services for both 1999 and 2020 with the respective value of 5.34 million US$ and 4.89 million US$. The group ESV change result shows that the provisioning, regulating, supporting, and cultural services were reduced by 22.24%, 21.2%, 8.49%, and 22.66%, respectively during the study period (Table 9).

**Table 9:** Annual ecosystem service value (US$ million per year) for ES category and individual function (ESF) and changes from 1999 to 2020.

ES Category	ESF	ESV_1999_	ESV_2020_	Change	% of change
Provisioning	Water supply	3.37	2.67	−0.70	−20.74
	Food production	11.25	8.81	−2.44	−21.72
	Raw material	4.81	3.89	−0.91	−19.01
	Genetic resource	84.55	65.48	−19.06	−22.55
	Total	103.97	80.85	−23.12	−22.24
Regulating	Gas regulation	0.70	0.59	−0.11	−15.96
	Climate regulation	114.34	89.14	−25.20	−22.04
	Disturbance regulation	3.69	2.87	−0.82	−22.24
	Water regulation	5.21	4.15	−1.06	−20.40
	Erosion control	19.38	15.83	−3.55	−18.33
	Water treatment	7.40	6.20	−1.20	−16.23
	Biological control	0.61	0.47	−0.14	−22.72
	Total	151.34	119.25	−32.09	−21.20
Supporting	Soil formation	0.80	0.66	−0.15	−18.40
	Nutrient cycling	0.64	1.13	0.50	77.98
	Pollination	1.69	1.33	−0.36	−21.22
	Habitat/refugee	2.21	1.77	−0.44	−20.00
	Total	5.34	4.89	−0.45	−8.49
Cultural	Recreation	48.33	37.37	−10.96	−22.68
	Cultural	0.12	0.10	−0.02	−16.75
	Total	48.45	37.47	−10.98	−22.66

Among 17 identified individual ESF; genetic resources, climate regulation, recreation, erosion control, and food production were dominant in the area. Climate regulation had the greatest individual ESV, with 114.34 million US$ in 1999 and 89.14 million US $ in 2020, followed by genetic resources, with 84.55 million US$ in 1999 and 65.48 million US$ in 2020. The lowest individual ESV was observed in cultural services for both 1999 and 2020. In summary, except for nutrient cycling, the individual ESV of SFBR reduced during the study period. In general, the total ESV of SFBR was reduced by 66.64 million US$ from 1999 to 2020. The sensitivity analysis found less than one for all LULC types for the entire study period (Appendix II), implying that the value of total ES assessed for SFBR is robust.

## 4 Discussion

### 4.1 LULC dynamics

Our result showed that LULC changed in the area. Natural forests and wetlands were converted to cropland, coffee plantations, tea plantation, and settlement. In contrast to the necessity for conservation, the country’s economic development policies that seek to boost national economic growth by attracting investors to areas like the SFBR have accelerated the LULC dynamics. The expansion of large-scale agricultural investment that is responsible for LULC conversion has become increasing in the last two decades. Since 2000, more than 35 large-scale (>1500 ha) coffee and tea investment projects started operation in the area [10]. In addition, the area is considered tea and a coffee-growing region and is prioritized for agricultural investment development. In line with this, numerous empirical studies reported the expansion of agricultural land at the expansion of natural forests in different parts of the country [16,51,52]. Moreover, the area’s rapid agricultural land expansion at the expense of forests and wetlands is aggravated by increased human population pressure & governmental resettlement programs [11]. In recent years, road construction and infrastructure developments have been carried out in the region, consequently, significant forest loss was discovered in the biosphere reserve. In conclusion, the spatial coverage of the forest and wetland decreased due to increased anthropogenic pressure to modify the natural ecosystem to boost economic return and support livelihood.

### 4.2 Impact of LULC on woody species diversity

The number of woody species in SFBR was higher than in some other moist Afromontane forests in Ethiopia. For example, 72 woody species were recorded in the Agama forest [30], 76 in the Wurg forest [27], and 44 in the Doske forest in Chencha [29]. Though SFBR hosted a significant number of woody species, LULC conversion attributed to the significant decline of woody species composition and structure. The forestland conversion to coffee plantations, cropland, home garden, and tea plantation reduced the number of species by 53.2%, 53.2%, 80.64%, and 96.8% respectively. Our observation was conveying that the impact of LULC conversion on biodiversity was higher than in other tropical forests [53,54]. Probably, this is because of the complete transition of intact natural forests to mono-cropping [10]. This is the worst and perhaps most distressing fact for all those concerned with nature conservation and sustainable uses.

Although the density was high, the average height of woody species in the coffee plantation was low, and intermediate height (middle story) was absent/minimal in the coffee plantation, indicating woody structural modification due to coffee cultivation. In line with this, [28] reported that the elimination of small woody species and subsequent replacement with coffee resulted in the dominance of coffee plants both in vertical and horizontal structures in the Berhane-Kontir and Harenna forests. The DBH distributions also convey that the great majority of woody species were clustered under the low DBH class, indicating the dominance of small trees/shrubs in the coffee plantation. Hence, coffee intensification and further modification of forest structure and composition affect both woody species diversity and coffee cultivation as shade trees become mature and reach the non-productive stage.

Although the woody species density of croplands of the study area by far more than similar land-use types in other parts of the country [55,56], the transition to cropland caused a 77.5% reduction in the woody species density. The average height of the remaining woody species in cropland was comparable to that of forests, and the mean DBH of cropland was higher than forests. Moreover, 40% of woody species were similar between forests and croplands. This is a good indicator of the existence of aged remnant trees in croplands. In the field research, we observed large-sized woody species such as *S. guineense, S. abyssinica, C. macrostachyus* in croplands. The critical challenge is that, since woody species regeneration is restricted in cropland, and existing old trees become perished, cropland will be devoid of trees shortly. This, in turn, leads to the local extinction of woody species including endemic and further affects the ecosystem’s resilience and stability.

The transition from forest to home garden caused an 86.85% reduction in woody species density. The similarity of woody species between forestland and the home garden was low (8%), indicating that native woody species are almost non-existent in the home garden. The IVI result showed that *Persea americana* and *Coffea arabica* were the dominant species of the home garden. This was caused by farmers' selection of multi-purpose trees for shade, food, and income generation.

As a mono-crop production system, tea plantations had a tremendous effect on the number of woody species and density. The transition from forest to a tea plantation for maximizing economic return induced the complete removal of indigenous woody species and replacement with exotic species. Due to their fast growth and ability to yield a high economic benefit immediately, exotic species were preferred over native ones. Our result disclosed that there was no similarity between the woody species in tea plantations and other LULC types. This is an indicator of the introduction of non-native (exotic) woody species in the area. Hence, the expansion of tea plantations in the region will not only influence native woody species diversity but also may lead to the expansion of exotic species. This, in turn, will harm ecosystem resilience and stability.

Woody species richness significantly varies with LULC types. The woody species richness of cropland, coffee plantation, home garden, and tea plantations was 65%, 65%, 80.64%, and 96.78% lower than forests, respectively. These results indicate that the transition from forest to non-forest land-use types has a tremendous effect on woody species richness. The number of woody species hosted in cropland, coffee plantations, and the home garden was lower than the similar land types in Ethiopia. For example, cropland hosted 42 woody species in northern Ethiopia [55], and 77 in East Shewa [56]. Similarly, numerous studies acknowledged the contribution of coffee agroforestry and home garden to biodiversity conservation in some parts of southern Ethiopia [[57], [58], [59]]. For example, 39 woody species were reported in home gardens in Gedeo [57], 46 in the Hawassa Zuria district [58], and 120 in the Sidama zone [59]. In this view, the contribution of non-forest land-use types to woody species richness was a minimum in SFBR.

The woody species diversity of non-forest land-use types was significantly lower than forestland. This is an indicator of the presence of a significant trade-off of woody species diversity due to LULC conversion in the study area. The lower diversity and evenness indices in non-forest land-use types were an indicator of the high abundance of one or a few woody species in the systems. Similarly, [28] showed the high abundance of a few species in coffee plantations. This implies that LULC conversion and transition to the mono-cropping system caused a reduction in woody species diversity and evenness in the area. If the “business as usual” approach continues, the diversity of woody species become impaired shortly, which would harm the production and productivity of currently preferred land-use types.

Although forest land conversion is attributed to a significant decline in biodiversity, the ruminant woody species in cropland, home gardens, and coffee plantations have conservation value in SFBR. Numerous woody species found in non-forest land-use types were either absent or rare in adjacent natural forests, indicating that non-forest land-use types were refugees for some conservation priority species. A similar result was reported in south-central parts of Ethiopia [60]. Although all converted land use types varied in their role to shelter conservation priority species, our result conveyed that the conservation effort should not be limited to forests; rather attention should also be given to the converted landscapes as they shelter conservation priory species.

### 4.3 Impact of LULC dynamics on ecosystem service value

The total ESV declined by 21.56% in the last two decades in SFBR. The overall ESV reduction has resulted from the expansion of settlement, coffee and tea plantations, and cropland in the area. Similar to this, other empirical studies in Ethiopia reported a progressive decline in total ESV over time [7,9,49,61]. During the study period, the highest total ESV was recorded for forests due to the large spatial coverage of natural forests in the area. However, the ESV of the forest reduced by 31% in 2020 as compared to its ESV in 1999. An increased LULC conversion pressure in the natural forests was responsible for the decline of total ESV over time in the area. On the other hand, the ESV of coffee plantations largely increased due to the spatial expansion of coffee cultivation in the area.

The ESV of all individual ES was reduced over time except for nutrient cycling. In contrast to our finding, most studies showed an increased ESV of food production [36,49], water supply [7,9], and biological control [37] in different parts of Ethiopia. The overall reduction of the majority of ecosystem services was closely associated with the loss of forests and wetlands over the study period. The positive change in ESV of nutrient cycling could be linked with the highest value assigned for non-forest land use types. Despite of declining trend of ESV, the SFBR had a huge contribution to climate regulation and genetic resource conservation. Hence, maintaining the ecosystem integrity through minimizing prevailing pressure on natural forests and wetlands of tropical rainforests could play a crucial role to sustain vital ecosystem services.

## 5 Conclusion

Our findings revealed that LULC change had a detrimental impact on the woody species diversity and associated ecosystem services and continued to alter biodiversity unless appropriate conservation and management measures are in place. LULC change-induced reduction in ESV is an indicator of ecological degradation in SFBR. Therefore, failure to resolve land use land cover change challenges will impede our effort for biodiversity conservation and sustainable use, limit the reliability of future projections and have repercussions on the conservation of threatened ecosystems. Although all types of LULC conversion have had an impact on woody species composition, structure, and diversity, shifting to tea/coffee monocropping plantations was a critical problem in the area. Alternative ways of maximizing resource use without compromising its sustainability, such as introducing payment for ecosystem services, would safeguard the few remaining threatened ecosystems. Although forestland conversion affects biodiversity, we found that some non-forest land-use types were refugees for some endemic and conservation-priority species. Therefore, conservation efforts should not be limited to forests; rather they should go hand in hand with non-forest land-use types.

## Author contribution statement

Yericho Berhanu: Conceived and designed the experiments; Performed the experiments; Analyzed and interpreted the data; Contributed reagents, materials, analysis tools or data; Wrote the paper.

Gemedo Dalle; Sintayehu W. Dejene; Girma Kelboro; Abebe Nigussie: Conceived and designed the experiments; Performed the experiments; Analyzed and interpreted the data; Wrote the paper.

## Funding statement

This work is supported by Movement for Ecological Learning and Community Action (MELCA Ethiopia), Bonga University, Africa Center of Excellence for Climate Smart Agriculture and Biodiversity Conservation at Haramaya University.

## Data availability statement

Data included in article/supplementary material/referenced in article.

## Declaration of competing interest

The authors declare that they have no known competing interests of conflict.

## Figures and Tables

**Table:** 

S.no	Scientific name	FL	CP	CL	Hg	TP
1	*Acacia brevispica* Harms	–	1.60	–	–	–
2	*Acanthus eminens* C.B.Cl	1.81	–	–	–	–
3	*Albizia grandibracteata* Taub.	5.45	–	–	–	–
4	*Albizia gummifera* (Gmel.) C. A. Sm.	8.87	14.82		6.59	
5	*Allophylus abyssinicus* (Hochst.) Radlkofer	5.71	1.52	–	–	–
6	*Allophylus macrobotrys* Gilg	5.84	–	–	–	–
7	*Apodytes dimidiata* E. Mey. ex Arn.	1.96	–	–	–	–
8	*Bersama abyssinica* Fresen.	5.18	–	2.35	–	–
9	*Brucea antidysenterica* J. F. Mill.	1.20	–	3.45	–	–
10	*Calpurnia aurea* (Ait.) Benth.	2.27	–	–	–	–
11	*Camellia sinensis*	–	–	–	–	195.61
12	*Canthium oligocarpum* Hiern	4.30	–	–	–	–
13	*Cassipourea malosana* (Baker) Alston	2.04	–	1.82	–	–
14	*Cayrotia* sp.	1.64	–	–	–	–
15	*Celtis gomphophylla* Bak.	0.76	–	–	–	–
16	*Celtis zenkeri* Engl.	4.75	–	–	–	–
17	*Chionanthus mildbraedii* (Gilg & Schellenb.) Stearn	11.30	–	–	–	–
18	*Clausena anisata* (Willd.) Benth.	4.12	–	–	–	–
19	*Coffea arabica* L.	4.25	117.11	3.32	74.79	
20	*Combretum paniculatum* Vent.	1.14	–	–	–	–
21	*Cordia africana* Lam.	6.67	27.23	3.43	22.12	
22	*Croton macrostachyus* Del.	12.85	9.09	17.21	–	–
23	*Cyathea manniana* Hook.	4.84	–	1.70	–	–
24	*Deinbollia kilimandscharica* Taub.	0.82	–	–	–	–
25	*Dombeya torrida* (J.F. Gmel.) Bamps	3.52	–	–	–	–
26	*Dracaena afromontana* Mildbr.	5.79	1.24	–	–	–
27	*Dracaena steudneri* Schw. ex Engl.	9.46	6.30	6.29	–	–
28	*Ehretia abyssinica* R. Br. ex Fresen.	0.90	2.75	10.48	–	–
29	*Ekebergia capensis* Sparrm.	–	1.28	2.19	24.61	–
30	*Elaeodendron buchananii* (Loes.) Loes.	–	2.37	–	–	–
31	*Erythrina brucei* Schweinf.	1.41	–	8.25	–	–
32	*Eucalyptus camaldulensis*	–	–	7.82	–	–
33	*Euphorbia ampliphylla* Pax	–	–	38.27	14.08	–
34	*Ficus* sp.	5.08	1.65	1.68	–	–
35	*Ficus sur* Forsssk.	–	3.61	–	3.79	–
36	*Ficus vasta* Vahl.	12.30	–	–	–	–
37	*Gouania longispicata* Engl.	1.46	–	–	–	–
38	*Grevillea robusta*	–	2.08	–	–	104.39
39	*Hallea rubrostipulata* (K. Schum.) Leroy	2.90	1.35	–	–	–
40	*Hibiscus* sp.	5.04	–	–	–	–
41	*Hippocratea africana* (Willd.) Loes.	2.22	–	–	–	–
42	*Hippocratea goetzei* Loes.	2.72	–	–	–	–
43	*Ilex mitis* (L.) Radlk.	–	2.13	9.08	20.34	–
44	*Jasminum abyssinicum* Hochst. ex Dc.	3.86	–	–	–	–
45	*Juniperus procera*	–	–	–	4.54	–
46	*Justicia schimperiana* T.Anders	2.00	–	–	–	–
47	*Keetia gueinzii* (Sond) Bridson	3.08	–	–	–	–
48	*Landolphia buchananii* (Hall. f.) Stapf	4.04	–	–	–	–
49	*Lepidotrichilia volkensii* (Gürke) Leroy	–	2.84	–	–	–
50	*Macaranga capensis* (Baill.) Sim	13.11	–	4.66	–	–
51	*Maesa lanceolata* Forssk.	11.76	–	10.41	–	–
52	*Mangifera indica*	–	1.26	–	–	–
53	*Maytenus arbutifolia* (A. Rich.) Wilczek	–	–	7.82	–	–
54	*Maytenus gracilipes* (Welw. ex Oliv.) Exell	5.93	–	37.19	–	–
55	*Millettia ferruginea* (Hochst.) Bak	–	31.69	9.96	8.88	–
56	*Olea capensis*	1.00	7.86	5.03	–	–
57	*Oxyanthus speciosus* DC.	1.99	–	–	–	–
58	*Pavetta abyssinica* Fresen	2.51	–	–	–	–
59	*Persea americana*	–	1.30	–	77.89	–
60	*Phoenix reclinata* Jacq.	2.50	2.87	–	–	–
61	*Phytolacca dodecandra* L’Hérit	0.76	–	1.66	–	–
62	*Pittosporum viridiflorum* Sims	3.58	–	–	–	–
63	*Polyscias fulva* (Hiern) Harms	–	–	4.67	–	–
64	*Pouteria adolfi-friederici* (Engl.) Baehni	5.58	8.44	–	–	–
65	*Prunus africana* (Hook. f.) Kalkm	–	1.85	–	–	–
66	*Prunus persica*	–	–	6.16	15.88	–
67	*Psychotria orophila* Petit	6.89	–	–	–	–
68	*Rhamnus prinoides* L’Herit.	1.15	–	–	–	–
69	*Ricinus communis* L.	2.10	–	–	–	–
70	*Rothmannia urcelliformis* (Hiern) Robyns	0.95	–	–	–	–
71	*Rubus steudneri* Schweinf.	3.47	–	–	–	–
72	*Sapium ellipticum* (Krauss.) Pax	6.78	–	1.82	–	–
73	*Schefflera abyssinica* (Hochst. ex A. Rich.) Harms	–	23.75	36.31	26.50	–
74	*Schefflera myriantha* (Bak.) Drake	28.19	–	–	–	–
75	*Sesbania sesban* (L.) Merrill	–	3.03	–	–	–
76	*Syzygium guineense* (Willd.) DC.	17.60	8.79	38.18	–	–
77	*Teclea nobilis* Del.	2.02	–	–	–	–
78	*Trema orientalis* (L.) Blume	11.23	5.18	–	–	–
79	*Trichilia dregeana* Sond.	2.99	5.01	–	–	–
80	*Trilepisium madagascariense* DC.	2.45	–	–	–	–
81	*Turraea holstii* Guerke	1.33	–	–	–	–
82	*Vepris dainellii* (Pich.-Serm.) Kokwaro	0.58	–	–	–	–
83	*Vernonia amygdalina* Del.	–	–	2.02	–	–
84	*Vernonia auriculifera* Hiern	–	–	16.79	–	–

**Table:** 

	1999		2020	
	%	CS	%	CS
Cropland VC ± 50%	0.35	0.0069	0.796192519	0.01592
Forest VC ± 50%	47.16	0.9432	41.43664103	0.82873
Coffee plantation VC ± 50%	1.35	0.027	6.587631899	0.13175
Tea plantation VC ± 50%	0.00	1E-05	0.021390275	0.00043
Wetland VC ± 50%	1.14	0.0229	1.157523498	0.02315
Settlement VC ± 50%	0.00	0	0	0

## Appendix I Importance Vegetation Index of Woody Species by LULC type in SFBR

*Note:* - is used to denote the absence of species occurrence, FL is forest, CP is coffee plantations, CL cropland, Hg is home garden and TP is Tea plantation.
